# Performance Improvement in Geographic Routing for Vehicular *Ad Hoc* Networks

**DOI:** 10.3390/s141222342

**Published:** 2014-11-25

**Authors:** Omprakash Kaiwartya, Sushil Kumar, D. K. Lobiyal, Abdul Hanan Abdullah, Ahmed Nazar Hassan

**Affiliations:** 1 Wireless Communications and Networking Research Lab, School of Computer and Systems Sciences, Jawaharlal Nehru University, New Delhi 110067, India; E-Mails: skdohare@yahoo.com (S.K.); lobiyal@gmail.com (D.K.L.); 2 Faculty of Computing, Universiti Teknologi Malausia (UTM), Skudai Johor 81310, Malaysia; E-Mails: hanan@utm.my (A.H.A.); nhahmed2@live.utm.my (A.N.H.)

**Keywords:** geographic distance routing, vehicular *ad hoc* networks, traffic environment, segment vehicle, link quality, degree of connectivity

## Abstract

Geographic routing is one of the most investigated themes by researchers for reliable and efficient dissemination of information in Vehicular *Ad Hoc* Networks (VANETs). Recently, different Geographic Distance Routing (GEDIR) protocols have been suggested in the literature. These protocols focus on reducing the forwarding region towards destination to select the Next Hop Vehicles (NHV). Most of these protocols suffer from the problem of elevated one-hop link disconnection, high end-to-end delay and low throughput even at normal vehicle speed in high vehicle density environment. This paper proposes a Geographic Distance Routing protocol based on Segment vehicle, Link quality and Degree of connectivity (SLD-GEDIR). The protocol selects a reliable NHV using the criteria segment vehicles, one-hop link quality and degree of connectivity. The proposed protocol has been simulated in NS-2 and its performance has been compared with the state-of-the-art protocols: P-GEDIR, J-GEDIR and V-GEDIR. The empirical results clearly reveal that SLD-GEDIR has lower link disconnection and end-to-end delay, and higher throughput as compared to the state-of-the-art protocols. It should be noted that the performance of the proposed protocol is preserved irrespective of vehicle density and speed.

## 1. Introduction

In the growing world economy, the number of vehicles is increasing day by day, thus making driving more risky and unsafe. The Global Status Report on Road Safety of the World Health Organization (WHO) summarized the statistics on road safety around the world. The report mentions 182 countries covering 99% population of the world. According to the report, the total number of road traffic deaths worldwide due to various traffic accidents on road is 1.24 million per year. The UN General Assembly declared the report as the basis for all the actions that would be taken for road safety during the entire decade 2011–2020 [1]. Further, the Delhi government's 12th five year development plan for transport department is based on a survey conducted by a group comprising RITES Ltd, MVA Asia and LDT in October, 2010. This survey demonstrates that the *per capita* trip rate has increased from 0.72 in 1981 to 0.87 in 2001 and it has been projected to increase to 1.2 by 2021. It further specified that Delhi Intra-City Motorized Person's trips are likely to rise from 11.7 million in 2007 to 17.4 million in 2021. An upsurge in Inter-City trip from 3.34 million per day in 2007 to 7.96 million per day has been predicted in 2021. This group has recommended the use of intelligent signaling system with Global Positioning System (GPS)- and Geographic Information System (GIS)-based applications to cope up with high degree of traffic population growth. The story of traffic population growth is similar for other capital cities of the world as well [2].

**Figure 1.**
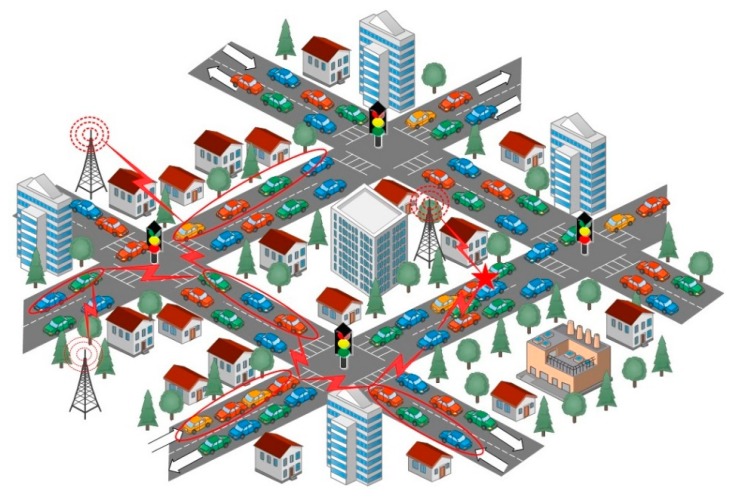
Vehicle-to-Vehicle (V2V) and Vehicle-to-Infrastructure (V2I) communication.

The aforementioned studies show that controlling both traffic congestion and vehicle accidents throughout the world is highly desirable. In order to achieve these goals, expensive sensors, radars, cameras and other state-of-the-art technologies have been currently incorporated into vehicles to improve safety and comfort during traveling [3]. Recently, Vehicle to Vehicle (V2V) and Vehicle to Infrastructure (V2I) applications have attracted more attention from industries and governments around the world because of their matchless potential to address safety and traffic congestion challenges at lower operational cost [4–6]. Both these applications (V2V and V2I) can also be used for commercial and vehicular infotainment [7,8]. In Figure 1, an example of V2V and V2I communication in Vehicular *ad hoc* Networks (VANETs) has been illustrated for the advancement of traffic control system and ease of travel in urban traffic environment.

VANETs have become a promising area of the research due to their potential to provide solutions for most of the traffic problems. VANETs are a contemporary concept, evolving as a new research area that incorporates three earlier communication research areas, namely *ad hoc* networks, wireless LAN, and cellular telephony [9]. VANETs are distinct from other kinds of *ad hoc* networks due to their hybrid network architectures, high speed vehicle movement, battery usage, self-organizing nature, distributed communication networks and wide range of new comfort applications [10]. VANETs employ a variety of advanced wireless technologies such as Dedicated Short Range Communications (DSRC) [11], which is an enhanced version of the Wi-Fi technology suitable for the VANET environment [12]. DSRC was developed to support data transfer in a rapidly changing communication environment, where time critical response and high data rates are desired. IEEE 802.11p is a wireless communication protocol especially designed for VANETs [13,14]. To incorporate Delay Tolerant Network capabilities in VANETs, a new network architecture has evolved. It is known as Vehicular Delay Tolerant Network (VDTN). The major characteristics of VDTNs, which are different from VANETs, are listed below. They are based on a store-and-carry-forward technique. They facilitate communication without end-to-end connectivity. They are suitable for non-real time applications where delay and reliability can be compromised up to some extent [15,16].

Efficient and reliable information distribution is one of the most challenging tasks in VANETs due to their highly dynamic network environment [17,18]. Recently, various geographic routing protocols have been suggested in the literature [19–24]. Geographic routing is also known as geocast routing or position based routing. The idea of geocast routing came from the Unix-to-Unix Copy (UUCP) Mapping project [25], which aimed at relating IP addresses with geographic locations by maintaining a database of geographic locations of each Internet hosts. The projects, Domain Name Server (DNS) encoding of geographic locations [26] and Internet Message Access Protocol Uniform Resource Locator (IMAP-URL) scheme [27], were also aimed at associating geographical locations of each host with host name. The above three projects made it possible to identify the geographical locations of in-coming data flows but directing out-going data flows to geographical locations was not possible. Geographic Routing was first suggested in [28] through the Cartesian routing that uses longitude and latitude information of node as address. In Cartesian routing, greedy forwarding technique has been used to forward packets to the neighbor nearest to the destination using geographical information of neighboring nodes.

Geographic routing or geocast routing is a variant of multicast routing. Information forwarding from a single source node to a group of destination nodes is known as multicast routing. In geographic routing, information is forwarded from single source node to a group of destination nodes within a specified geographical region that is known as Zone of Relevance (ZOR) [29]. In multihop forwarding of geographic routing, the concept of Zone of Forwarding (ZOF) has been used to improve reliability in forwarding. All the nodes within the area specified by ZOF are considered as next hop nodes of the current forwarder node [30]. Geographic routing is one of the most preferred choices for information dissemination in vehicular traffic environment. The preference can be attributed to the fact that the on-road vehicles can be grouped into geographical regions and traffic information can be shared considering the groups as geocast regions. The other benefits of geographic routing are as follows. It is not restricted to infrastructure support. It is flexible for most ITS applications.

The three most preferred and recent geographic routing protocols are Voronoi diagram-based Geographic Distance Routing (V-GEDIR) [31], Junction-based Geographic Distance Routing (J-GEDIR) [32] and Peripheral node-based Geographic Distance Routing (P-GEDIR) [33]. These state-of-the-art protocols have been investigated and considered for comparative analysis in this paper. V-GEDIR selects the next hop nodes in a Voronoi region towards the destination and J-GEDIR selects junction nodes within the transmission range as next hop vehicles. P-GEDIR vertically divides the circular coverage region into two half-circular sections and selects all the border nodes as next hop vehicles from the half-circular section towards the destination. These protocols reduce the size of the forwarding region for selecting a smaller number of next hop vehicles. The performance of these protocols starts degrading with higher vehicle density and speed in the reduced forwarding region. Further, these protocols select border vehicles as next hop vehicle to minimize the number of hop count from source to destination, but the border vehicles have higher outage probability during forwarding due to the high vehicle speed in vehicular traffic environment. In urban environments, the quality of links is highly affected by obstacles such as buildings, trees, *etc*. These protocols ignore the link quality consideration in next hop vehicle selection.

In this paper, the Segment vehicle, Link quality and Degree of connectivity based Geographic Distance Routing (SLD-GEDIR) protocol is proposed. The protocol chooses a small region at the boundary of the circle formed by transmission range as radius and the source node as the center of the circle. The small region has been referred to as segment area. Vehicles present in the segment area are called segment vehicles. The selection of NHV depends on segment vehicle, one-hop link quality, and degree of connectivity. The key components of the present study are as follows: (1) determining the number of segment vehicles, which means finding all the vehicles that belong to the segment area of current forwarding vehicle; (2) prediction of one-hop link quality using packet error rate; (3) determining the degree of connectivity of NHV with segment vehicles. The NHV with the highest degree of connectivity has been considered as most reliable forwarder. These components of the proposed protocol have been analyzed and validated through analytical results. The proposed protocol has been simulated in ns-2. A comparative study has been carried out between SLD-GEIDR and other state-of-the-art protocols: V-GEDIR, J-GEDIR and P-GEDIR. The rest of the paper has been organized as follows: in Section 2, early and recent research works on position-based and geographic routing protocols have been summarized. A description of the mathematical formulations and details of the conceptualization of SLD-GEDIR protocol is provided in Section 3. The analytical and simulation results have been discussed in Section 4. The conclusions of the study are presented in Section 5.

## 2. Related Work

Position-based routing might be a better choice in VANETs for a number of applications; e.g., collision warnings, alerts messages, advertisements, *etc*. This is precisely because of their ability to handle variation in the position of vehicles [34]. Still, there are issues in position-based routing that need to be addressed. Position-based routing can be improved by incorporating location error probability and its possible solution. Location information can be easily changed by attackers as all the vehicles broadcast their location information to neighboring vehicles. Flooding in multi-hop routing increases the network overhead, therefore, an efficient algorithm that selects a minimum number of next forwarding vehicles is needed [35]. Geographic forwarding protocols select the shortest route that may suffer from a higher packet error rate due to poor link quality. The uneven distribution of vehicles on the roads makes route selection more complex; e.g., the shortest path in terms of geographic distance may experience more frequent path disconnections. The dynamic and rapidly changing topology of vehicular networks can cause frequent communication disconnections among vehicles. The frequent path disconnection is the most important issue in designing routing protocols for VANETs. A few protocols use vehicle density information to select routes, but the inaccuracy of statistical data may cause routes to be incorrectly computed. The link quality depends on the shadowing phenomena caused by obstacles in the path of transmission.

**Figure 2.**
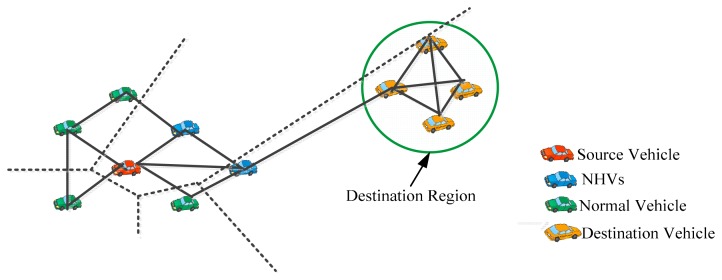
NHVs selection using V-GEDIR.

Voronoi Diagram based Geographic Distance Routing (V-GEDIR) [31], is an extension of GEDIR. In this paper the authors have presented a loop-free position-based routing algorithm. In the V-GEDIR algorithm, NHVs are selected as vehicles whose Voronoi region either covers or cuts the expected destination zone. This method can be used for both routing and geocasting. An example of the NHV selection process using V-GEDIR is shown in Figure 2.

**Figure 3.**
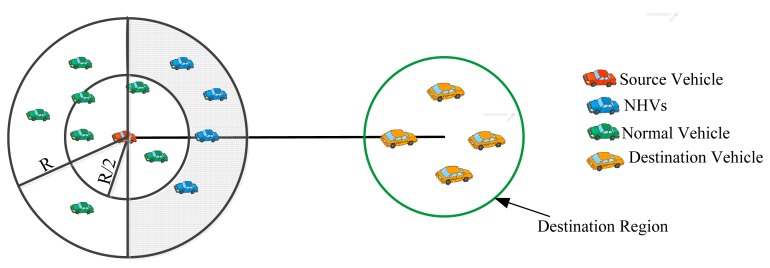
NHVs selection using P-GEDIR.

In V-GEDIR, node mobility is not considered during the formation of a Voronoi diagram, which limits the applicability of the approach in highly mobile networks such as VANETs. In [32], the authors have suggested a Junction-based Geographic Routing (J-GEDIR) protocol for VANETs. In the protocol, an on-road vehicle passing through a junction point, closest to the destination and making smallest angle with the source vehicle has been selected as next hop vehicle. The greedy forwarding based on distance selects border nodes as NHVs that degrade the performance of the protocol in a high speed vehicular environment due to non-inclusion of link quality in the consideration. In Peripheral node-based Geographic Distance Routing (P-GEDIR) [33], all vehicles present inside the circular strip of width R/2 (R is the transmission range of a vehicle) towards the destination are referred to as peripheral vehicles. Peripheral vehicles are considered as NHVs. In a high speed vehicular environment such as a highway, selecting peripheral nodes as NHVs limits the performance of P-GEDIR due to the high outage probability of peripheral nodes. These protocols are not suitable for dense VANETs which do not include link quality into the consideration and their performance starts degrading with the increasing speed of vehicles. An example of NHVs selection process using P-GEDIR is shown in Figure 3.

A Multi-Metric map aware routing protocol for VANETs has been suggested in [36]. The protocol has used four metrics: distance, density, trajectory and bandwidth, for making routing decisions. In the protocol, fixed and predefined weights are assigned to each of the considered metrics that is one of the most noticeable deficiencies of the protocol considering highly dynamic network environments in VANETs. The Adaptive Connectivity Aware Routing (ACAR) [37] protocol addressed rapidly changing topology and frequent network disconnections by dynamically selecting an optimal route with the best transmission quality. It is based on real time vehicle density estimation and collection. Real time vehicle density estimation is one of the more challenging and highly error prone tasks. Due to its direct impact on the reliability of route selection, the protocol performance is affected by the amount of errors occurred in this density estimation. Moreover, the protocol performance is suspect in low vehicle density environment because of high dependency of the protocol on vehicle density. A Free standing Position Based Routing (FPBR) for highway traffic environment has been presented in [38]. Different modules have been developed to deal with different highway scenario issues such as high vehicle speed, propagation conditions, *etc*. The protocol is strictly limited the the highway traffic environment only considering its scenario-based modules and functionalities.

By predicting the next position of vehicles in the future, a routing algorithm for V2V communication in urban traffic environment has been suggested in [39]. The routing algorithm, Grid-based Predictive Geographical Routing (GPGR) reduces the breakage of links during forwarding by estimating the future position of NHVs. The grid-based position estimation of NHVs limits the applicability of the protocol due to the development of modern road networks that does not follow a grid-based structure. Relative Position-Based Message Dissemination (RPB-MD) in VANETs has been presented in [40]. In RPB-MD, the destination is identified using an anonymous addressing model based on the relative positions of vehicles. Once the destination is identified, directional greedy broadcast routing is used to forward messages by selecting a group of upstream vehicles for holding messages and improving forwarding reliability. Content-based routing has been investigated in [41] by temporarily grouping vehicles for multicasting in VANETs. In the Spatio-Temporal Multicast Routing protocol (SMRP), a multicast group is created utilizing subscription information and clustering. SMRP always forwards single copy messages to the whole group of subscribers. The applicability of the subscription-based information dissemination approach is mostly confined to the areas of advertising and entertainment in VANETs.

The concept of VDTN has been introduced in [15,16] and a geographic routing protocol, GeoSpray for VDTN has been presented in [42]. GeoSpray utilizes a hybrid forwarding approach between multiple-copy and single-copy forwarding schemes. First, it starts with a multiple-copy scheme and spreads a limited number of packets in the network. Then, it changes to a forwarding scheme that utilizes the inter-vehicle contacts to ensure the delivery of packets to the vehicle closer to the destination. In order to improve resource utilization, it periodically clears the delivered bundles across the network. The waiting time analysis of packets is missing in this work. This limits the protocol's applicability in various ITS applications requiring lower bounda in packet delivery time. A mobicast routing protocol with carry-and-forward has been presented in [43]. Mobicast supports non-emergency comfort applications. It uses Zone of Relevance (ZOR)-based forwarding technique. The protocol's applicability is limited to only entertainment applications due to its strict policy of using carry-and-forward despite the availability of NHVs. The Geographic Delay Tolerant Network routing with Navigator (GeoDNT + Nav) [44] has been proposed for urban environmenta. It enhances the standard greedy and recovery mode forwarding by exploiting the vehicular mobility and on-board vehicular navigation systems to efficiently deliver packets, even in partitioned networks.

In [45], authors have presented a new VANET approach based on two key assumptions. First, geo-anycast functionality is not required by the applications and second, geographic unicasting is not needed when IP-based unicasting is provided. It combines the features of Internet Protocol version 6 (IPv6) and non-IP geographical networking. The approach is useful only when the infrastructure required for IPv6 is deployed throughout the network in terms of mobile towers. A hybrid position-based forwarding technique for VDTN has been suggested in [46]. It combines the position-based and store-carry-forward routing approaches and utilizes vehicle direction information in holding or forwarding messages. In VDTN, utilizing vehicle speed in transportation of message is one of the important points in routing that is not addressed in [46]. Lower bound analysis of delay has not been performed in the work that limits the applicability of the protocol, especially in VDTNs. Readers are advised to consult [47], where an evaluation study of geocast protocols and their possible applications in VANETs is presented.

## 3. SLD-GEDIR Protocol

In VANET traffic environment, geographic- and position-based routing protocols perform better, particularly for ITS application warning systems [47]. In both types of protocols, packets are forwarded to all the vehicles within a specified geographical region. This increases forwarding overhead, wastes bandwidth, and results in looping problems. Researchers have focused their attention on solving these problems [48]. Due to the constraints of vehicular traffic environment such as high variations in vehicle speeds, unstable channel conditions, unreliable link quality, high collision probability, *etc*., the design of efficient routing protocols in VANETs has been a challenging task. The design of the SLD-GEDIR protocol is a sincere effort to address the abovementioned problems of geographic routing by closely analyzing some of these constraints. SLD-GEDIR is based on the following three main concepts:

- Segment Vehicles
- One-Hop link quality Prediction
- Degree of connectivity of NHV

The following assumptions have been made in the design of SLD-GEDIR.

— Hello (beacon) control messages are exchanged to identify current neighbors.
— Vehicles are equipped with GPS receiver, road/street digital map and sensors.
— Dedicated Short Range Communication (DSRC) protocol is used for vehicle to vehicle communication [12].
— No other communication infrastructure is required.
— MAC and Physical layer Protocols are considered to be functioning properly.
— Maximum Transmission range for all the vehicles is considered equal.

The following subsections provide the mathematical formulations and details of the conceptualization of SLD-GEDIR.

### 3.1. Segment Vehicles

**Figure 4.**
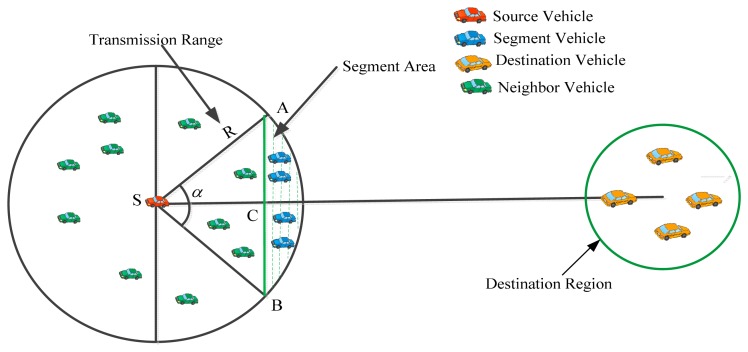
Segment vehicles and neighbor vehicles.

In SLD-GEDIR, each vehicle has a predefined maximum transmission range. All the vehicles within the transmission range of a specified vehicle are called neighboring vehicles of the specified vehicle. Each vehicle sends a hello message to all its neighbors to collect their speed, location, direction and time information. Segment vehicles are those vehicles that are present within the segment area. The segment area of a vehicle *S* is shown in Figure 4 as the area with dashed lines.

The segment area *S_area_* of a vehicle *S* can be calculated as given in Equation (1):

(1) $$\begin{array}{c} S_{\text{area}}=\mathrm{Area of Sector}\text{SAB}-\text{Area of}\Delta \text{SAB}\\ S_{\text{area}}=\frac{\alpha }{360{}^{\circ}}\pi R^{2}-\frac{L}{4}\sqrt{4R^{2}-L^{2}} \end{array}$$

where *L* = *AB, M* = *SC and* $\alpha =2\times \tan^{-1}\left(\frac{L}{2M}\right)$. By substituting these assumptions, the Equation (1) can be further simplified as given in Equation (2):

(2) $$S_{\text{area}}=\left(R^{2}\times \tan^{-1}\left(\frac{L}{2M}\right)\right)-\left(\frac{L}{4}\times \sqrt{4R^{2}-L^{2}}\right)$$

#### 3.1.1. Probabilistic Analysis of the Presence of Vehicles in the Segment Area in Non-Shadowing Environments

In this section, the circular transmission range of the vehicles has been considered. To setup one-hop links for forwarding packets, the sender vehicle needs to find at least one NHV in the segment area. The presence of vehicles in the segment area depends on three parameters vehicle density *λ*, sector angle *α*, and transmission range of vehicle *R*. One of the main objectives of SLD-GEDIR is to analyze the impact of the parameters *λ, α* and *R* on the probability of finding at least one vehicle in the segment area. To achieve this objective, different values are assigned to *α* in increasing order until a vehicle is found in the segment. The vehicles are moving in two-dimensional network area and availability of vehicles in the network area follows a Poisson distribution with vehicle density *λ*. Given the mean density of vehicles in the network, the number of vehicles present in the segment area is calculated using a Poisson distribution. Further, the arrival of each vehicle is independent.

SLD-GEDIR uses the position information of vehicles for routing. The position information of a vehicle is represented by both *x* and *y* coordinates on a plane. Therefore, a 2D network model has been considered, as used in [31,33,49]. Various studies have been done to find the optimal transmission range for VANET environments. These studies clearly reveal that the transmission range requirement decreases with increasing vehicle density. The high density vehicular traffic environment requires a smaller transmission range to avoid simultaneous conflicting transmissions resulting in collisions [50,51]. Therefore, a smaller value of *R* (250 *m*) has been considered for the analysis.

If *X* is the random variable which represents number of vehicles present in the segment area, then the probability $P_{S_{\text{area}}}^{\text{NSE}}\left(X=n\right)$ of the presence of *n* vehicles in the segment in a non-shadowing environment can be calculated as given by Equation (3):

(3) $$P_{S_{\text{area}}}^{\text{NSE}}\left(X=n\right)=\frac{{\left(\lambda \times S_{\text{area}}\right)}^{n}\times e^{-\left(\lambda \times S_{\text{area}}\right)}}{n!}$$

By substituting the value of *S_area_* given in Equation (2), the Equation (4) is obtained:

(4) $$P_{S_{\text{area}}}^{\text{NSE}}\left(X=n\right)=\left[\frac{{\left[\lambda \left\{\left(R^{2}\times {\text{tan}}^{-1}\left(\frac{L}{2M}\right)\right)-\left(\frac{L}{4}\times \sqrt{4R^{2}-L^{2}}\right)\right\}\right]}^{n}}{n!}\right]\times e^{-\lambda \left\{\left(R^{2}\times {\text{tan}}^{-1}\left(\frac{L}{2M}\right)\right)-\left(\frac{L}{4}\times \sqrt{4R^{2}-L^{2}}\right)\right\}}$$

By substituting *n* = 0, the probability $P_{S_{\text{area}}}^{\text{NSE}}\left(X=0\right)$ of presence of no vehicle in the segment area in a non-shadowing environment can be calculated as expressed in Equation (5):

(5) $$\begin{array}{c} P_{S_{\text{area}}}^{\text{NSE}}\left(X=0\right)=\left[\frac{{\left[\lambda \left\{\left(R^{2}\times {\text{tan}}^{-1}\left(\frac{L}{2M}\right)\right)-\left(\frac{L}{4}\times \sqrt{4R^{2}-L^{2}}\right)\right\}\right]}^{0}}{0!}\right]\times e^{-\lambda \left\{\left(R^{2}\times {\text{tan}}^{-1}\left(\frac{L}{2M}\right)\right)-\left(\frac{L}{4}\times \sqrt{4R^{2}-L^{2}}\right)\right\}}\\ P_{S_{\text{area}}}^{\text{NSE}}\left(X=0\right)=e^{-\lambda \left\{\left(R^{2}\times {\text{tan}}^{-1}\left(\frac{L}{2M}\right)\right)-\left(\frac{L}{4}\times \sqrt{4R^{2}-L^{2}}\right)\right\}} \end{array}$$

The probability $P_{S_{\text{area}}}^{\text{NSE}}\left(X\geq 1\right)$ of the presence of at least one vehicle in the segment area in a non-shadowing environment can be expressed as given in Equation (6):

(6) $$P_{S_{\text{area}}}^{\text{NSE}}\left(X\geq 1\right)=1-e^{-\lambda \left\{\left(R^{2}\times {\text{tan}}^{-1}\left(\frac{L}{2M}\right)\right)-\left(\frac{L}{4}\times \sqrt{4R^{2}-L^{2}}\right)\right\}}$$

#### 3.1.2. Impact of Non-Circular Transmission Range on the Probabilistic Analysis of the Presence of Vehicles in a Segment Area (Shadowing Environment)

**Figure 5.**
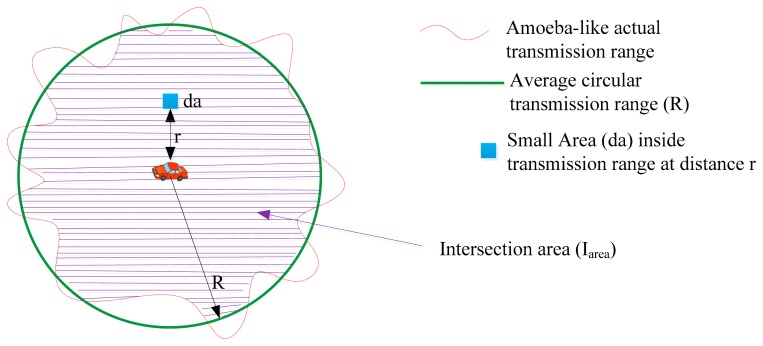
Non-circular transmission range shape due to shadowing.

The probabilistic analysis of the presence of vehicles in the segment area in vehicular traffic environments can be made more realistic by using a non-circular transmission range. The assumption of a non-circular transmission range is the basis for integrating the shadowing model with the segment area. The circular shape of the transmission range assumed for ideal conditions is transformed into an amoeba-like shape due to the shadowing effect in vehicular traffic environment. The actual transmission range varies in different directions due to the effect of shadowing [52] on the received power (*cf.* Figure 5), which is given in Equation (7):

(7) $$PW_{r}=PW_{t}\left\{10\log_{10}C-10\omega \log_{10}\left(\frac{d}{d_{0}}\right)-\tau \right\}$$

where, *C* is a constant that represents channel attenuation and antenna characteristics, *ω* denotes the path loss exponent, *d* and *d*_0_ represents the distance between vehicles and the reference distance for antenna respectively. The term *τ* is a Gaussian random variable.

As shown in Figure 5, a small incremental area *da* at distance *r* can be used to find the intersection area *I_area_* between the circular transmission range and then amoeba-like transmission area. In *I_area_* received power, *PW_r_* always remains above the minimum required power *PW_min_* to decode a signal. *I_area_* can be calculated as given in Equation (8):

(8) $$I_{\text{area}}=\frac{1}{\pi R^{2}}\int_{0}^{2\pi }\int_{0}^{R}p\left(PW_{r}\left(r\right)\geq PW_{\text{min}}\right)r\;dr\;d\theta$$

where *PW_r_* (*r*) represents the received power in area *da* at distance *r*. Log-normal distribution accurately models the variation in received power due to shadowing [53]. Therefore, using log-normal distribution, the probability of received power at distance *r* being greater than *P_min_* is represented by *p*(*PW_r_* (*r*) ≥ *PW_min_*) and can be calculated as given in Equation (9):

(9) $$p\left(PW_{r}\left(r\right)\geq P_{\text{min}}\right)=Q\left(\frac{PW_{\text{min}}-\left(PW_{t}+10\log_{10}C-10\omega \log_{10}\left(r/d_{0}\right)\right)}{\sigma_{\tau }}\right)$$

where, $Q\left(t\right)=\int_{t}^{\infty }\frac{1}{\sqrt{2\pi }}e^{-\frac{x^{2}}{2}}dx$, and $\sigma_{\tau }^{2}$ represents the variance of *τ*.

By using Equation (9), the *I_area_* can be simplified as given in Equation (10):

(10) $$I_{\text{area}}=\frac{2}{R^{2}}\int_{0}^{R}Q\left(x+y\log\frac{r}{R}\right)r\;dr$$

where $x=\frac{PW_{\text{min}}-PW_{r}^{avg}\left(R\right)}{\sigma_{\tau }}$ and $y=\frac{10\omega \log_{10}\left(e\right)}{\sigma_{\tau }}$. Here, $PW_{r}^{avg}\left(R\right)$ represents the average received power at distance *R*. Equation (10) can be further simplified as given in Equation (11):

(11) $$I_{\text{area}}=Q\left(x\right)+e^{\left(\frac{2-2xy}{y^{2}}\right)\times Q\left(\frac{2-2xy}{y}\right)}$$

By assuming $PW_{\text{min}}=PW_{r}^{avg}\left(R\right)$, Equation (1) can be expressed as given in Equation (12):

(12) $$I_{\text{area}}=\frac{1}{2}+e^{\left(\frac{2}{y^{2}}\right)\times Q\left(\frac{2}{y}\right)}$$

By using *I_area_*, Equation (6) can be modified to find the probability, $P_{S_{\text{area}}}^{SE}$ of selecting at least one vehicle in the segment area under shadowing environment as expressed in Equation (13).

(13) $$P_{S_{\text{area}}}^{SE}\left(X\geq 1\right)=\left[1-e^{-\lambda \left\{\left(R^{2}\times \tan^{-1}\left(\frac{L}{2M}\right)\right)-\left(\frac{L}{4}\times \sqrt{4R^{2}-L^{2}}\right)\right\}}\right]\times \left[\frac{I_{\text{area}}}{\pi R^{2}}\right]$$

### 3.2. One-Hop Link Quality Prediction under Non-Shadowing and Shadowing Environment Conditions

In SLD-GEDIR, each vehicle possesses the city road map and knows the past movement of the other vehicles. Therefore, the quality of links between sender vehicles and segment vehicles can be estimated. To predict the link quality, first, the two-ray ground reflection model has been considered. The shadowing model which is more suitable for vehicular environment has also been considered in the last paragraph of this section. The received power between two vehicles according to the two-ray ground reflection model is given in Equation (14):

(14) $$PW_{r}=\frac{PW_{t}G_{t}G_{r}H_{t}^{2}H_{r}^{2}}{d_{\text{link}}^{4}l}$$

where *PW_r_* and *PW_t_* are the received and transmitted power respectively, *G_t_* and *G_r_* are the antenna gain of transmitter and receiver, *H_t_* and *H_r_* are the height of transmitter and receiver antenna, *d_link_* is the link distance between vehicles and *l* is the system loss. In practice, received power is not the only parameter used to determine link quality. Signal-to-noise ratio *SNR_link_* of the link, which is given in Equation (15), is also an important parameter:

(15) $${\text{SNR}}_{\text{link}}=\frac{a^{2}PW_{r}}{PW_{th}+a^{2}PW_{\text{inf}}}$$

where, *a* is the amplitude of the fading channel with Rayleigh distribution, *PW_th_* is the thermal noise power, and *PW_inf_* represents the interference power. By using Binary Phase Shift Keying (BPSK) modulation, the Bit Error Rate (*BER_link_*) of the link is given in Equation (16) [54]:

(16) $$BER_{\text{link}}=\frac{1}{2}\left(1-\sqrt{\frac{{\text{SNR}}_{\text{link}}}{1+{\text{SNR}}_{\text{link}}}}\right)$$

The Packet Error Rate *PER_link_* over a one hop link is computed as expressed in Equation (17):

(17) $${\text{PER}}_{\text{link}}=\left\{1-{\left(1-{\text{BER}}_{\text{link}}\right)}^{L}\}+\{f_{m}\left(n\right)\right\}$$

where, *L* is the length of the packet in bits and *f_m_*(*n*) is the vehicle mobility function. The first term of Equation (17) *i.e.*, {1− (1 − *BER_link_*)*^L^*} represents a general packet error rate caused by link failure that does not include failure due to mobility. The second term of Equation (17) *i.e.*, {*f_m_*(*n*)}is the heuristic function used to predict packet error probability due to sudden directional changes of the vehicles. The function *f_m_*(*n*) has a past knowledge component and a future heuristic component. The function *f_m_*(*n*) is expressed in Equation (18):

(18) $$f_{m}\left(n\right)=1-\left(\frac{1}{g\left(n\right)+h\left(n\right)}\right)$$

where, *g*(*n*) represents the number of directional changes taken by the vehicle in the past and *h*(*n*) denotes the number of directional changes expected by the vehicle in future to reach the destination using the shortest available route. As soon as the total number of directional changes {*g*(*n*) + *h*(*n*)} increases, the value of vehicle mobility function 0 ≤ *f_m_* (*n*) ≤ 1 also increases. In case of transmission failure, a packet is retransmitted. A packet can be successfully transmitted at least once in N retransmissions. The probability of successful transmission can be expressed as $\sum_{i=0}^{N}\left(1-{\text{PER}}_{\text{link}.}\right){\text{PER}}_{\text{link}}^{i}$. Each retransmission effort has been denoted by *i*. Therefore, the Packet Error Rate ${\text{PER}}_{\text{link}}^{N}$ over one-hop link with *N* retransmissions can be expressed by Equation (19):

(19) $${\text{PER}}_{\text{link}}^{N}=1-\sum_{i=0}^{N}\left(1-{\text{PER}}_{\text{link}.}\right)PER_{\text{link}}^{i}$$

Packet Error Rate ${\text{PER}}_{\text{link}}^{N}$ of a path with *N* retransmissions in each one-hop link and that is made up of *k* number of one-hop links can be computed as given in Equation (20):

(20) $${\text{PER}}_{\text{path}}^{N}=1-{\left(1-PER_{\text{link}}^{N}\right)}^{k}$$

The ${\text{PER}}_{\text{link}}^{N}$ can be calculated using either Equation (19) or (20) with *k* = 1. One-hop link quality prediction between vehicles in vehicular traffic environment can be made more realistic by incorporating the impact of shadowing on received power *PW_r_* as shown by Equation (7). The model of the impact of shadowing on ${\text{PER}}_{\text{link}}^{\text{N}}$ can be analyzed using Equation (20) by substituting the value of *PW_r_* given in Equation (7) into Equation (15).

### 3.3. Impact of Degree of Connectivity of NHVs on Successful Transmission

**Figure 6.**
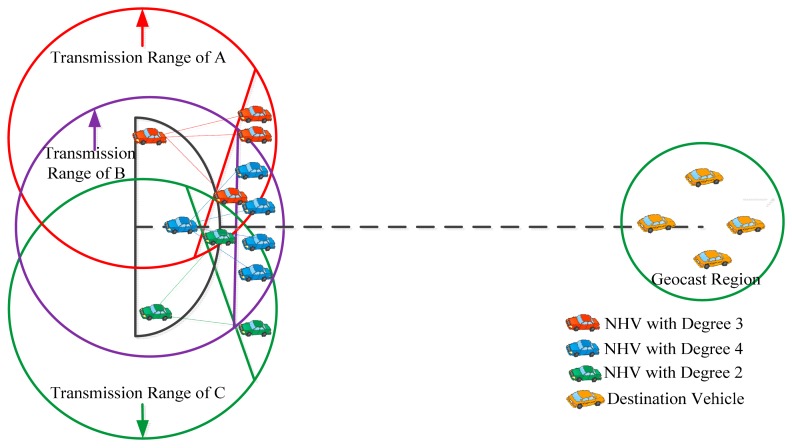
NHV selection based on degree.

The degree of connectivity of NHVs is defined as the number of links between the NHV and its neighbors within a respective segment area (*cf.* Figure 6). The degree of connectivity of NHVs is used to identify a reliable forwarding vehicle from among the segment vehicles. A segment vehicle having the highest degree of connectivity in its respective segment area will have higher probability of finding a better NHV, even if some of the segment vehicles undergo directional changes. In SLD-GEDIR, each vehicle has information about its position, degree of connectivity, average speed, and time. Vehicles exchange this information among neighbors. The information about the degree of connectivity plays an important role in making efficient routing decisions in the proposed protocol.

The probability of finding an NHV *P_NHV_* in the segment area can be expressed as given in Equation (21) (*cf.* Figure 4, for *CABC* and *SAB*):

(21) $$\begin{array}{cc} P_{\text{NHV}} & =\frac{\text{Number of vehicles in segment area CABC}}{\text{number of vehicles in sector area SAB}}\\ & =\frac{\lambda \left\{\left(R^{2}\times \tan^{-1}\left(\frac{L}{2M}\right)\right)-\left(\frac{L}{4}\times \sqrt{4R^{2}-L^{2}}\right)\right\}}{\lambda \left(R^{2}\times \tan^{-1}\left(\frac{L}{2M}\right)\right)} \end{array}$$

By simplification of Equation (21), Equation (22) is obtained:

(22) $$P_{\text{NHV}}=1-\left\{\frac{\frac{L}{4}\times \sqrt{4R^{2}-L^{2}}}{R^{2}\times \tan^{-1}\left(\frac{L}{2M}\right)}\right\}$$

### 3.4. SLD-GEDIR Algorithm

In this section, a geographic routing algorithm for VANETs is presented. It is aimed at reducing the forwarding overheads and improving the NHV selection criteria. The algorithm considers segment vehicles, one-hop link quality, and degree of connectivity of NHVs, direction of vehicle, speed, and sector angle for making a precise NHV selection decision. The SLD-GEDIR algorithm is presented below:


**Algorithm: SLD-GEDIR**
**Notations** *CFV*: Current Forwarding Vehicle *SSV*: Set of Segment Vehicle (potential Next Forwarder Vehicle) *ODV*: Original Destination Vehicle *OSV*: Original Source Vehicle *NHV*: Next hop Vehicle SONV: Set of One hop Neighbor Vehicles *V^i^*: Individual *i^th^* Vehicle *d_i_*: Degree of connectivity of *i^th^* segment vehicle *α*: Sector angle used for segment area calculation
$Q_{i}^{\text{link}}$: Quality of one-hop link of *i^th^* segment vehicle**Input**
*R, λ, L, M, P_t_, P_r_, G_t_, G_r_, H_t_, H_r_, l, d_link_***Process** **1. initialization**  *CFV* = *OSV*  *SSV* = *ϕ*  *NHV* = *ϕ*  *SONV* = *ϕ*  *α* = 50° **2.**
*SONV* = {neighbors of CFV} **3. if** (*ODV* ∈ *SONV*) **then**  Send the packet to *ODV* using available direct link  **exit** **4. else**  **while** (*SSV* ⩵ *ϕ*)   a. **Calculate** segment area S_area_ using equation (2)   b. SSV = {Vehicles in S_area_}   c. **if** (*SSV* ⩵ *ϕ and α* < 180°)    *α* = *α* + 100°   d. **Else**    Wait for random amount of time    *α* = 50°  **end while** **end if** **5. for** each vehicle *V_i_* ∈ *SSV*  Calculate packet error rate 
$PER_{\text{link}}^{N}\left(i\right)$ using equation (19)
$Q_{i}^{\text{link}}=\left(1-PER_{\text{link}}^{N}\left(i\right)\right)$  **end for** **6. for** each vehicle *V_i_* ∈ *SSV*   Calculate degree of connectivity *d_i_*  **end for** **7.**
$Q_{j}^{\text{link}}+d_{j}=Max\left\{Q_{1}^{\text{link}}+d_{1},Q_{2}^{\text{link}}+d_{2}+Q_{3}^{\text{link}}+d_{3},\ldots Q_{n}^{\text{link}}+d_{n}\right\}$ **8.**
*HV* = *V_j_* **9. transmit** the packet to *NHV* and *NHV* = *CFV* **10. exit****Output:**
*NHV* = (*V_j_*)


#### Explanation of Phases of SLD-GEDIR

**Figure 7.**
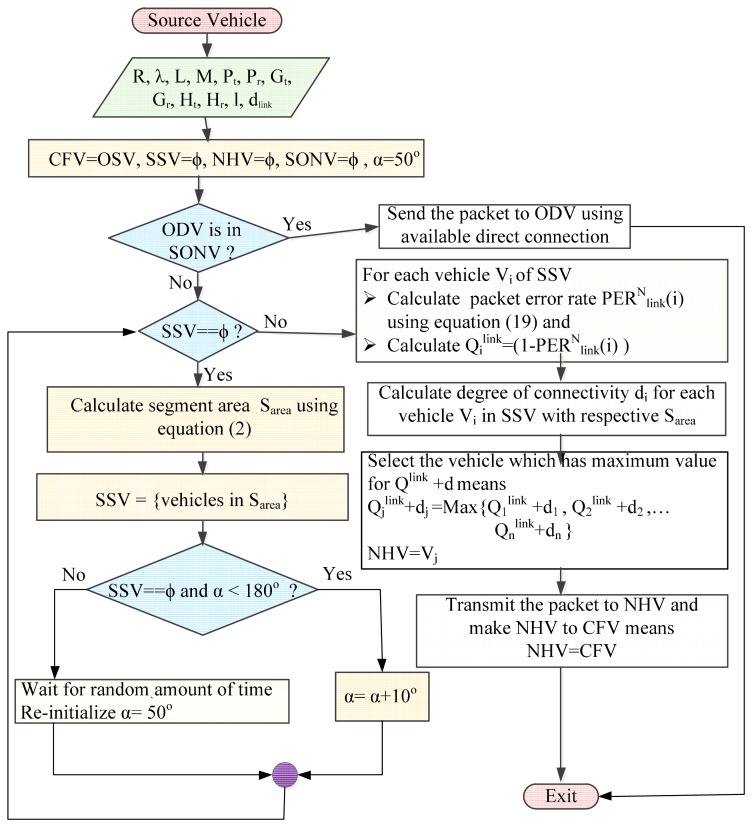
Flowchart of the SLD-GEDIR algorithm.

As soon as a vehicle receives a packet for forwarding towards a destination vehicle, it uses steps 1–10 of the SLD-GEDIR algorithm. In the 1st step, initialization of variables is performed. In the 2nd step, the SONV set is assigned information about neighbors of the current vehicle. In the 3rd step, CFV checks whether ODV is in the SONV set. If ODV is found in the SONV set, CFV transmits a packet to the destination vehicle using the available direct link. In the case ODV where it is not in the SONV set, the algorithm executes step 4, in which a set of segment vehicles (SSV) with smallest possible sector angle is determined. The quality of one hop links associated with each vehicle of SSV is computed in the 5th step. In the 6th step, degree of connectivity of each vehicle in the SSV is calculated. In the 7th and 8th steps, an NHV is determined to forward the packet further. In the 9th step, the packet is delivered to the NHV and the NHV becomes the CFV for the next hop forwarding. Steps 1 to 10 are used at each hop until the packet is delivered to the ultimate destination. To facilitate the understanding of logical flow of steps in the algorithm, a flowchart is presented in Figure 7.

## 4. Empirical Results

In this section, the experimental results obtained to analyze the performance of the proposed protocol and its elementary mathematical formulation has been provided. The section is broadly divided into two sections. In the first Section 4.1, analytical results have been obtained to validate the mathematical formulations. Simulation results are discussed in the Section 4.2.

### 4.1. Analytical Result

**Figure 8.**
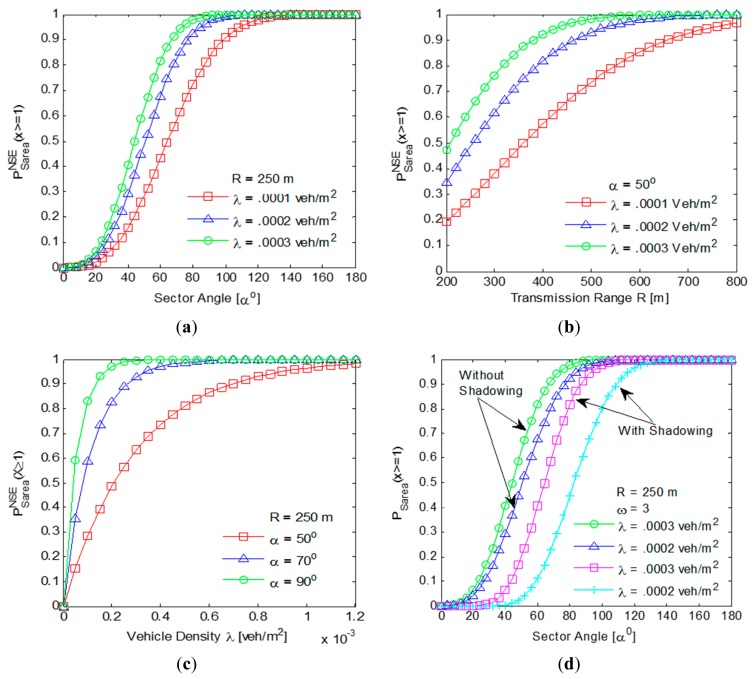
Impact of parameters in $P_{S_{\text{area}}}^{\text{NSE}}\left(X\geq 1\right)$ and $P_{S_{\text{area}}}^{SE}\left(X\geq 1\right)$.

In this section, analytical results have been obtained using MATLAB to analyze the impact of parameter changes on the mathematical formulations considered. The set of values of various parameters required to obtain the results have been mentioned in the individual plots themselves. The impact of parameter changes on $P_{S_{\text{area}}}^{\text{NSE}}\left(X\geq 1\right)$ and $P_{S_{\text{area}}}^{SE}\left(X\geq 1\right)$ is shown in Figure 8.

From the results depicted in Figure 8a, it can be clearly observed that for the offset angle *α* = 50°, the probability $P_{S_{\text{area}}}^{\text{NSE}}\left(X\geq 1\right)$ of presence of at least one vehicle in the segment area in a non-shadowing environment is 0.7 for a vehicle density λ = 0.0003 vehicles/m2. The value α = 50° has been considered as a minimum threshold to analyze the performance of SLD-GEDIR. The results depicted in Figure 8b clearly indicate that for each of the vehicle densities considered, the probability $P_{S_{\text{area}}}^{\text{NSE}}\left(X\geq 1\right)$ of the presence of at least one vehicle in the segment area in a non-shadowing environment is equal to or greater than 0.7 for the transmission range of 500 m or above. This result has been used to analyze the performance of SLD-GEDIR. The results in Figure 8c show that for sector angle *α* = 50°, the probability $P_{S_{\text{area}}}^{\text{NSE}}\left(X\geq 1\right)$ of the presence of at least one vehicle in the segment area in a non-shadowing environment is about 0.7 for a vehicle density *λ* = 0.0003 vehicles/m^2^. Moreover, for any particular value of the vehicle density considered, the probability $P_{S_{\text{area}}}^{\text{NSE}}\left(X\geq 1\right)$ increases with the increase in sector angle. For example, at *α* = 90°, the probability $P_{S_{\text{area}}}^{\text{NSE}}\left(X\geq 1\right)$ achieves the maximum value of approximately 1.0 for *λ* = 0.0003 vehicles/m^2^. The impact of shadowing on the probability *P_Sarea_* (*X* ≥ 1) of the presence of at least one vehicle in the segment area has been shown in the results depicted in Figure 8d. It clearly reveals that the impact of shadowing is high for a smaller sector angle (≤ 40°) but with the increase in sector angle (≥ 60°) the impact is reduced drastically.

**Figure 9.**
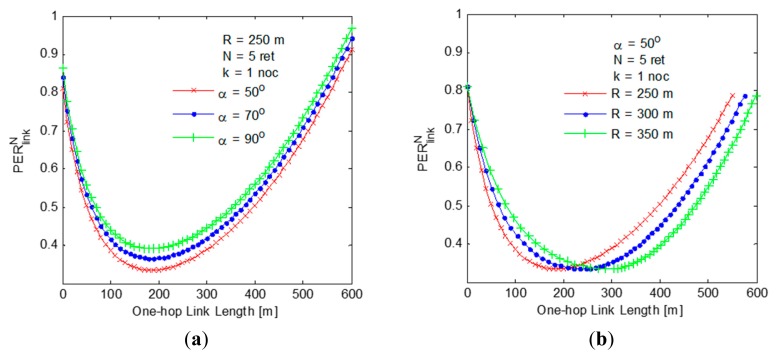

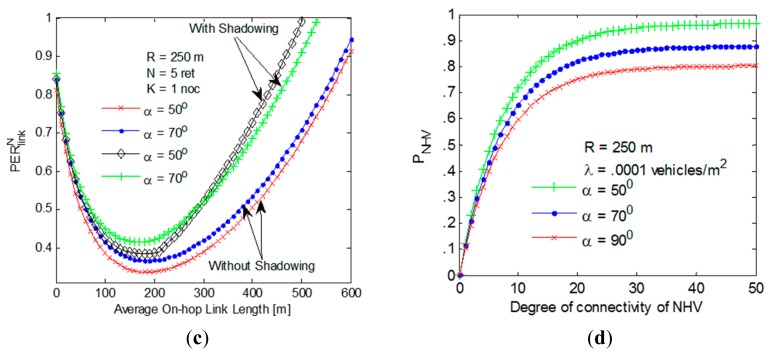
Impact of parameters on $PER_{\text{link}}^{N}$ (**a**) and (**b**) in non-shadowing environment, (**c**) in shadowing environment and (**d**) impact of parameters on *P_NHV_*.

The results depicted in Figure 9a show that $PER_{\text{link}}^{N}$ is lowest with a link length of 200 m for each values of sector angle that has been considered. This observation has been used for the selection of NHVs with the best quality link. The results also reveal that the vehicles belonging to the border area are not the best NHV choice as considered in the state-of-the-art protocols since the probability of going out of transmission range is higher for border vehicles. This outcome has been further validated by the other results given in Figure 9b. The results shown in Figure 9b clearly convey that for a better quality one-hop link, the NHV must be a vehicle present well-inside the segment area but not at the border of the segment area. For example, when *R* = 250 *m* is considered, the length of the best quality one-hop link is found to be approximately 200 *m*.

The results in Figure 9c show the impact of shadowing on $PER_{\text{link}}^{N}$. It can be clearly observed that the impact of shadowing increases drastically as soon as link length becomes larger than 200 *m*. As soon as the length of one-hop link approaches towards *R* = 250 *m*, $PER_{\text{link}}^{N}$ increases faster due to higher probability of border vehicles going out of range. The results depicted in Figure 9d show that the probability of finding an NHV increases with the increase in degree of connectivity. Moreover, it also suggests that smaller sector angles give better performance with less number of next hop vehicles. This can be attributed to the fact that the probability of forwarding towards the destination becomes higher with smaller sector angle.

### 4.2. Simulation Result

In this section, the outcome of the simulations carried out to analyze the performance of the SLD-GEDIR protocol in handling frequent changes in network topology due to the high mobility of vehicles and in managing dense vehicular network in an urban traffic environment are presented. In the simulation, end-to-end delay, throughput, and one-hop link disconnections in the network have been computed. Results obtained for SLD-GEDIR algorithm are compared with three state-of-the-art protocols: P-GEDIR, J-GEDIR and V-GEDIR.

#### 4.2.1. Simulation Environment

The SLD-GEDIR algorithm has been simulated using the NS-2.34 network simulator. The mobility model generator for vehicular networks (MOVE) has been used to generate a realistic mobility model and a realistic urban traffic environment. MOVE is built on the top of an open-source micro-traffic simulator, Simulation of Urban Mobility (SUMO). Most of the essential features of the vehicular traffic environment such as number of roads, number of lanes in each road, number of flows in each lane, number of junctions, traffic lights in a particular junction, speed of vehicles, probability of turning left or right of a vehicle at a particular junction etc. have been setup and implemented utilizing the two main modules of MOVE, namely the road map editor and vehicle movement editor. The mobility trace generated by MOVE with the help of SUMO, has been directly used in NS-2. Two types of vehicular traffic environment, High Speed and High Density has been considered to evaluate SLD-GEDIR. In a high speed traffic environment, the performance of the three SLD-GEDIR modules, namely segment vehicle, one-hop link quality and degree of connectivity in the segment area have been analyzed. In a high density traffic environment, the analysis focuses on verifying whether the three modules of SLD-GEDIR increase forwarding overheads with increasing vehicle density. A set of four horizontal and four vertical roads crossing each other and thus making sixteen junction points at equal distance is used as simulation area. Each road has double lanes. Important simulation parameters have been summarized in Table 1. The simulations have been performed after setting the network and traffic flow with the considered parameter values. In each simulation run the source vehicle and geocasting region is selected randomly from two pre-specified different junctions that remain same for all the 30 simulation runs. An average of 30 different simulation runs has been taken for each value used in the result analysis.

#### 4.2.2. Simulation metrics

The following routing metric are used to compare the performance of SLD-GEDIR with the state-of-the-art protocols:

End-to-End delay: End-to-end delay is the time taken by a packet to travel across network from source to destination and *vice versa*. It is the addition of transmission delay, propagation delay and processing delay for each of the links between sources to destination. The statistical formula used to calculate end-to-end delay in terms of millisecond can be expressed as given in Equation (23):

(23) $$\text{End}-to-\text{end delay}\left(ms\right)=\frac{\sum_{i=1}^{30}\sum_{j=1}^{PR}\left(ST_{i,j}-RT_{i,j}\right)}{PR\times 30}$$

where *PR* denotes the total number of packets received at destination, *ST_i_*_,_*_j_* is the sending time of *j^th^* packet in *i^th^* simulation, and *RT_i_*_,_*_j_* is the receiving time of *j^th^* packet in *i^th^* simulation.

Throughput: Throughput is the number of messages that have been delivered successfully from source to destination per unit time. It is measured in terms of bits per second. VANETs in highway traffic environments have been considered as a sparse network due to the availability of less forwarding options in highways. This is because volume of inter-city traffic is not very high as other modes of transportation are also available. The urban traffic environment has been considered as a dense network due to a high volume of traffic making a large number of forwarding options available in an urban environment [20]. The statistical formula used to calculate throughput in terms of kbps can be expressed as given in Equation (24):

(24) $$\text{Throughput}\left(\text{kbps}\right)=\frac{\sum_{i=1}^{30}\left(PS-PL\right)}{30}\times \frac{512}{1024\times 600}$$

where, *PS* denotes the number of packets sent from the source vehicle and *PL* represents the number of packets lost during forwarding.

One-hop Link Disconnection: The failure of message transportation during one-hop communication is defined as one-hop link disconnection. This metric shows the reliability of the hop-to-hop link establishment scheme. In other words, it verifies the quality of the NHV selection process. The statistical formula used to calculate one-hop link disconnection in terms of percentage can be expressed as given in Equation (25):

(25) $$\text{One}-\text{hop link disconnection}\left(\%\right)=\left\{\overset{\left(\sum_{i=1}^{30}\frac{RD}{PS}\right)}{\;}/30\right\}\times 100$$

where, *RD* represents total number of router drop for a packet from source to destination in *i^th^* simulation run and *PS* denotes number of packets sent in *i^th^* simulation run.

#### 4.2.3. Results

In this section, the simulation results obtained for SLD-GEDIR algorithm with 95% confidence interval have been presented. The section has been divided into three sub-Sections A, B and C. In sub-Section A, the impact of vehicle speed on the performance of SLD-GEDIR has been analyzed. In sub-Section B, the analysis of effect of vehicle density on the performance of SLD-GEDIR has been provided. In sub-Section C, a map-based performance evaluation of the proposed protocol has been presented.

##### A. Impact of Vehicle Speed

**Figure 10.**
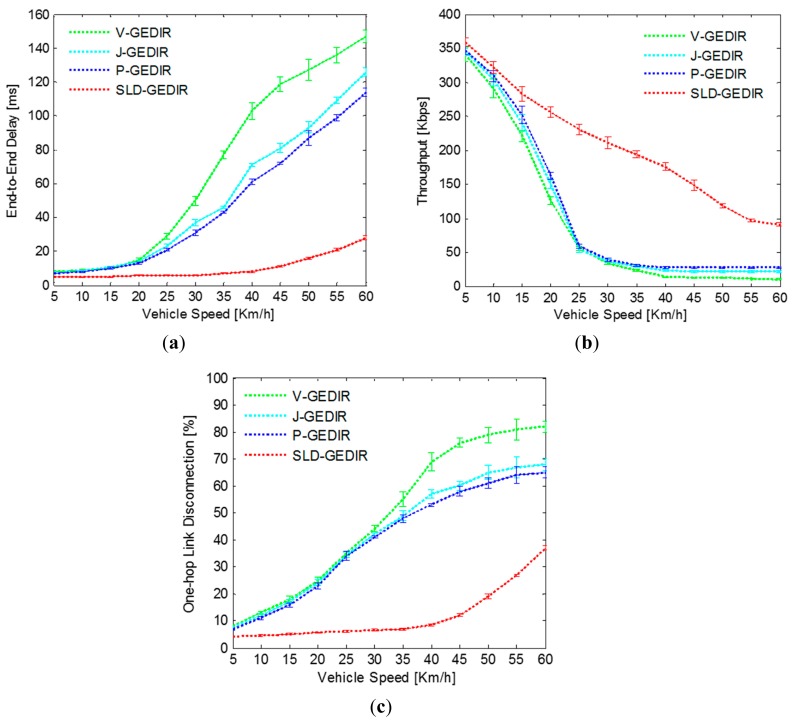
Impact of speed in (**a**) end-to-end delay; (**b**) throughput and (**c**) one-hop link disconnection.

In the simulation, end-to-end delay, throughput and one-hop link disconnections have been computed for different vehicle speed values. Vehicle speed has been varied from 5 – 60 Km/h to analyze the performance of SLD-GEDIR and compare it with the state-of-the-art protocols. The result depicted in Figure 10a shows that for vehicle speed in the range 5 – 30 Km/h, end-to-end delay remains constant and is nearly 6 *ms* for SLD-GEDIR protocol. Further, end-to-end delay is significantly reduced for SLD-GEDIR. This can be attributed to the fact that the NHV selection process has been made unambiguous towards the most probable direction of destination in SLD-GEDIR. The performance of J-GEDIR and P-GEDIR starts degrading at a speed above 20 Km/h. The reason behind this is that J-GEDIR selects the farthest junction vehicles as next hop vehicles and P-GEDIR selects border vehicles as next hop vehicles. The farthest junction vehicles and border vehicles are prone to go out of transmission range frequently due to the high mobility of the traffic environment. The performance of all the state-of-the-art protocols is approximately same for the speed range of 5 – 20 Km/h. The performance of V-GEDIR degrades at a higher rate as the vehicle speed goes above 20 Km/h because it does not consider the speed of vehicles for selecting a forwarding vehicle. Thus the proposed protocol outperforms the compared state-of-the-art protocols.

In Figure 10b, the results show the comparison of throughput between SLD-GEDIR and those of other protocols under consideration for varying speed. The results demonstrate that the throughput of SLD-GEDIR decreases linearly with increasing vehicle speed. However, the throughput decreases sharply for the state-of-the-art protocols. This can be attributed to the fact that the state-of-the-art protocols need to re-discover one-hop links more frequently because of high rate of link breakage with increasing vehicle speed. The link selected by SLD-GEDIR is based on various quality computations that make it more reliable. Therefore, SLD-GEDIR always outperforms the considered state-of-the-art protocols. The comparison result of one-hop link disconnection between SLD-GEDIR and the state-of-the-art protocols has been shown in Figure 10c. It can be clearly observed that one-hop link disconnection of SLD-GEDIR is nominal in comparison with the state-of-the-art protocols with the speed range of 5–40 Km/h. The reason behind this is that the degree of connectivity and the one-hop link quality prediction selects the most reliable NHV in the proposed protocol. However, the one-hop link disconnections suddenly increase at speeds above 40 Km/h because the NHV selection process could not be completed within the available time. P-GEDIR is the next best performer in terms of number of one-hop link disconnections compared to J-GEDIR and V-GEDIR. The one-hop link disconnections in P-GEDIR and J-GEDIR start growing rapidly at speeds above 20 Km/h because the forwarding technique used in these protocols does not consider speed information in NHV selection. V-GEDIR performs the worst as compared with other protocols for all speed ranges due to its larger hop count from source to destination region. Thus, the performance of SLD-GEDIR is better than the compared state-of-the-art protocols in terms of one-hop link disconnections.

##### B. Impact of Vehicle Density

**Figure 11.**
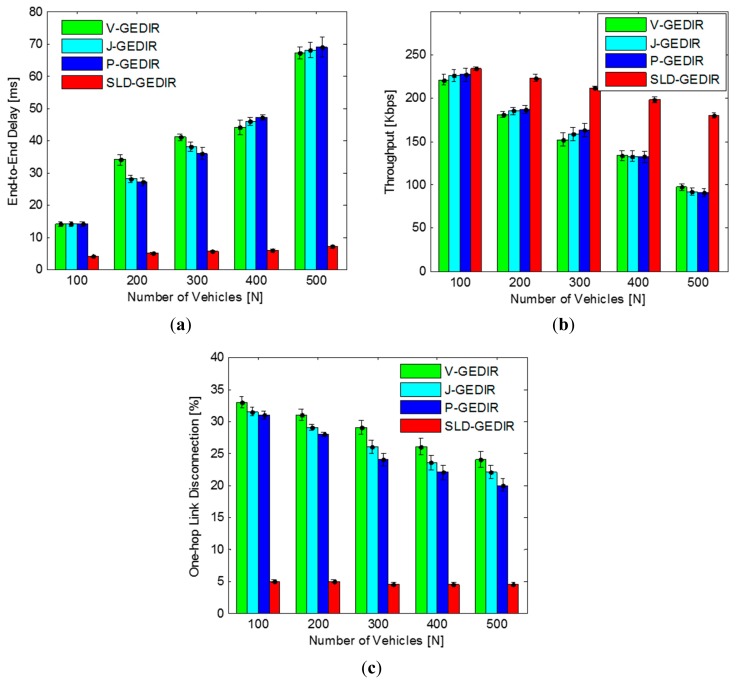
Impact of density in (**a**) end-to-end delay; (**b**) throughput and (**c**) one-hop link disconnection.

In this simulation, to analyze the impact of vehicle density on the performance of the proposed protocol, the number of vehicles was varied from 100 to 500. The results depicted in Figure 11a indicate that the SLD-GEDIR protocol has lowest end-to-end delay in comparison with the state-of-the-art protocols. The impact of vehicle density on end-to-end delay of the proposed protocol is insignificant due to its unique NHV selection process. The state-of-the-art protocols show higher end-to-end delays since they select more than one NHV. It is also noteworthy that the state-of-the-art protocols show nearly equal end-to-end delays since the protocols minimize the forwarding area and select all the vehicles within the forwarding region as NHVs. Thus, it is clearly noted that SLD-GEDIR outperforms other considered state-of-the-art protocols in terms of end-to-end delay with increasing density. The results depicted in Figure 11b indicate that the throughput of the proposed protocol is nearly equal to the considered state-of-the-art protocols with 100 vehicles in the segment area, but, as soon as the vehicle density increases, the throughput of the state-of-the-art protocols decreases rapidly with higher rates as compared to the proposed protocol. This is due to the fact that the proposed protocol almost eliminates duplication of packets whereas in case of state-of-the-art protocols, duplication of packets increases with the increase in vehicle density. Therefore, SLD-GEDIR performs better than the state-of-the-art protocols in terms of throughput.

The results are depicted in Figure 11c. It clearly shows that the number of one-hop link disconnections in SLD-GEDIR is lower as compared to the considered state-of-the-art protocols. It is also noteworthy that the link disconnection of the proposed protocol remains the same with increasing vehicle density whereas in case of state-of-the-art protocols link disconnections decrease marginally with increasing vehicle density. This can be attributed to the fact that the proposed protocol always selects a single NHV whereas in case of state-of-the-art protocols the number of NHVs increases with increasing vehicle density. The rate of one-hop link disconnection is 5% for the proposed protocol whereas it varies from 22% to 33% in the case of state-of-the-art protocols. The state-of-the-art protocols show approximately equal link disconnections for each vehicle density considered. Thus, it can be noted that the proposed protocol shows lower link disconnection as compared to the state-of-the-art protocols.

##### C. Map Based Analysis

**Figure 12.**
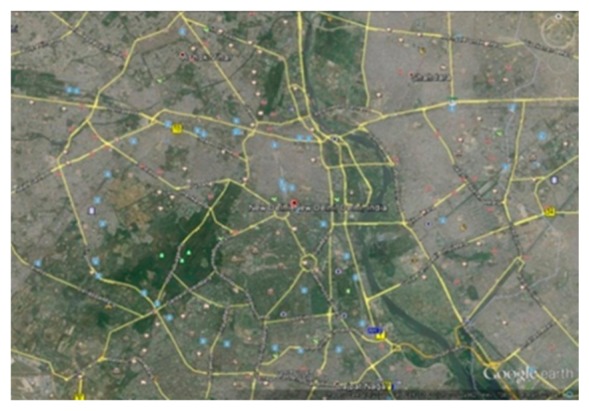
The city map of New Delhi, India.

**Figure 13.**
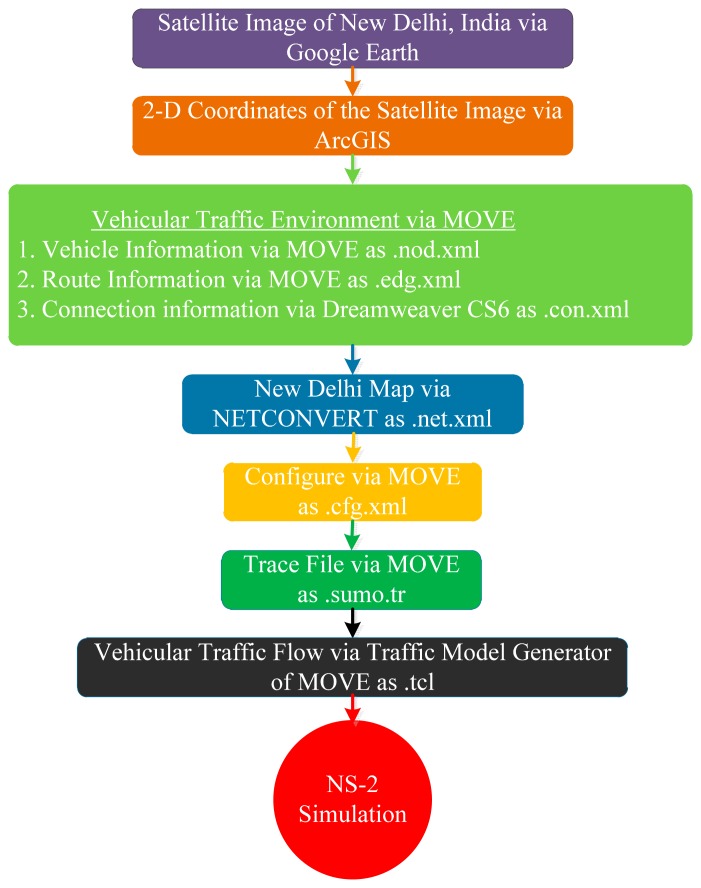
The Map-based simulation steps.

In this section, the performance of SLD-GEDIR has been analyzed considering the road network of New Delhi (India). A satellite image of the city (*cf.* Figure 12) is obtained *via* Google Earth software and it imported in ArcGIS 10.2.2 for the assignment of the road network coordinates. The city map is input to MOVE that integrates network information with the map. Thereafter, configuration and trace files have been generated and ultimately the vehicular traffic flow in the New Delhi Map has been produced to analyze the performance of the proposed protocol in a high speed and low vehicle density traffic environment. The complete process of map-based simulation has been depicted in Figure 13.

**Figure 14.**
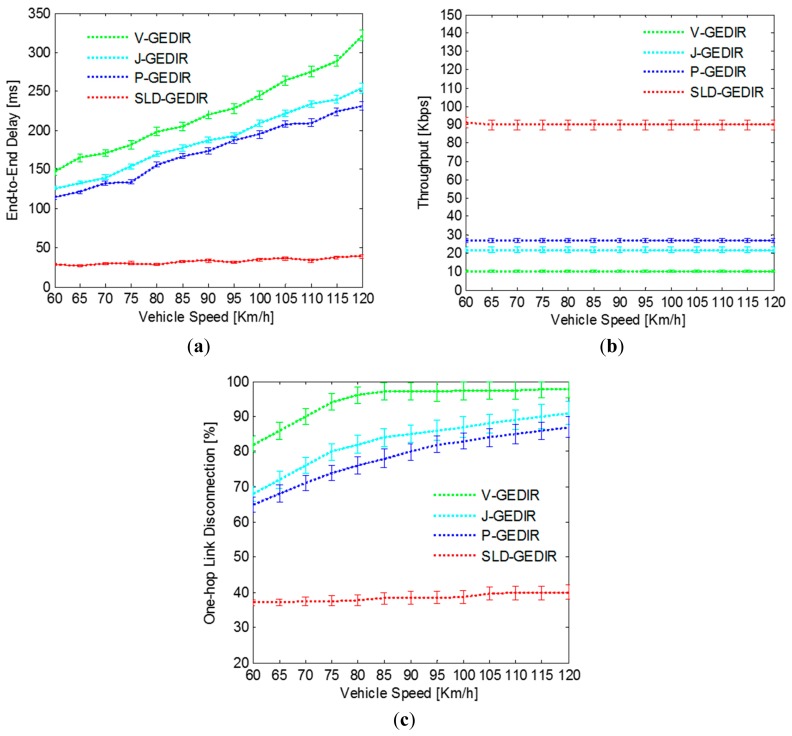
Map-based analysis (**a**) end-to-end delay; (**b**) throughput and (**c**) One-hop link disconnection.

The results depicted in Figure 14a show the comparison of end-to-end delay between SLD-GEDIR and the state-of-the-art protocols obtained through map-based simulation. It can be clearly observed that the end-to-end delay of the proposed protocol is below 50 *ms* for each of vehicle speed considered, whereas for all the considered state-of-the-art protocols, the end-to-end delay is above 100 *ms*. The difference in end-to-end delay can be attributed to the fact that the proposed protocol uses speed information in NHV selection whereas all the considered state-of-the-art protocols do not utilize speed information for the same consideration. A throughput comparison between SLD-GEDIR and the state-of-the-art protocols has been given in Figure 14b. The proposed protocol shows approximately 90 *Kbps* stable throughput as compared to the stable throughput below 30 *Kbps* of the state-of-the-art protocols. The reason behind the higher throughput of the proposed protocol in comparison with the state-of-the-art protocols is its single copy multihop forwarding technique which reduces network load and increases throughput. The stable nature of throughput of all the considered protocols is due to the lower vehicle density traffic environment considered in the simulation resulting in intermittently connected traffic environment. The results in Figure 14c show the comparison of one-hop link disconnection between SLD-GEDIR and the state-of-the-art protocols. The one-hop link disconnection of the proposed protocol is below 40% whereas it is above 65% in case of the state-of-the-art protocols. The difference in one-hop link disconnections is due to the link quality prediction of the proposed protocol utilized in NHV selection that effectively reduces one-hop link disconnections as compared to the state-of-the-art protocols. The state-of-the-art protocols did not consider link quality in their NHV selection process. Thus, the performance of SLD-GEDIR is better as compared to the state-of-the-art protocols in terms of end-to-end delay, throughput and one-hop link disconnection in the map-based simulation.

## 5. Conclusions

In this paper a geographic distance routing protocol for vehicular traffic environments called SLD-GEDIR has been proposed and simulated using NS-2. The performance of the protocol has been evaluated in terms of end-to-end delay, throughput, and one-hop link disconnection, and compared with the state-of-the-art protocols. An analysis of simulation results leads to the following conclusions: the impact of speed and density of vehicles on SLD-GEDIR in terms of end-to-end delay is nominal. SLD-GEDIR has lower end-to-end delay as compared to the state-of-the-art protocols. The throughput of SLD-GEDIR decreases linearly with increasing speed and decreases gradually with increasing density of vehicles. However, the throughput of the state-of-the-art protocols decreases sharply as the speed and density of the vehicles increases. Further, it was found that one-hop link disconnections in SLD-GEDIR are lower as compared to the state-of-the-art protocols. Moreover, the impact of vehicle density and speed on SLD-GEDIR is insignificant in terms of one-hop link disconnection. From the analysis of the results obtained through simulation it is clear that SLD-GEDIR is more suitable for vehicular traffic environments as compared to the state-of-the-art protocols. In the future research, authors will explore the idea of using Particle Swarm Optimization (PSO)-based NHV selection and Cache Agent (CA)-based forwarding in SLD-GEDIR.

## Figures and Tables

**Table 1.** Simulation Parameters.

**Parameters**	**Values**
Simulation area	1500 × 1000 *m*^2^
Simulation time	600 *s*
Number of vehicle	100 − 500
Vehicle speed	1.4 − 16.7 *m/s* (5 − 60 *Km/h*)
Transmission range	250 *m*
Packet senders	30
Traffic type	*CBR*
Packet size	512 *bytes*
Packet type	*UDP*
Ifqlen	50
CBR rate	6 *Packets/s*
Channel type	*Wireless*
Propagation model	*Shadowing*
Antenna model	*Omni directional*
MAC protocol	*IEEE* 802.11*p*
MAC data rate	5 *Mbps*
Query period	3 *s*
Hello timeout	1 *s*
Frequency	5.9 *GHz*
Routing protocols	SLD-GEDIR, state-of-the-art protocols

## References

[1] World Health Organization (2013). Global Status Report on Road Safety 2013: Supporting a Decade of action.

[2] Govt. of NCT of Delhi. An Approach to 12th Five Year Plan: Transport Department. http://www.delhi.gov.in/wps/wcm/connect/DoIT_Planning/planning/important+links/an+approach+;to+12th+five+year+plan+%282012-17%29.

[3] Emmelmann M., Bochow B., Kellum C. (2010). Vehicular Networking: Automotive Applications and Beyond.

[4] Hartenstein H., Laberteaux K. (2010). Vehicular Applications and Inter-Networking Technologies.

[5] Hu B., Gharavi H. (2011). Joint Vehicle-Vehicle/Vehicle-Roadside Communication Protocol for Highway Traffic Safety. Int. J. Veh. Technol..

[6] Huang Z., Singh G. A Communication Protocol for a Vehicle Collision Warning System.

[7] Gerla M., Kleinrock L. (2011). Vehicular networks and the future of the mobile internet. Comput. Netw..

[8] Di W., Yingrong B., Jie L. (2014). Cooperative Downloading by Multi vehicles in Urban VANET. Int. J. Distrib. Sens. Netw..

[9] Karagiannis G., Altintas O., Ekici E., Heijenk G., Jarupan B., Lin K, Weil T. (2011). Vehicular Networking: A Survey and Tutorial on Requirements, Architectures, Challenges, Standards and Solutions. Commun. Surv. Tutor..

[10] Al-Sultan S., Al-Doori M., Al-Bayatti A., Zedan H. (2014). A comprehensive survey on vehicular *Ad Hoc* network. J. Netw. Comput. Appl..

[11] US Department of Transportation “DSRC Standards: What's New?” ITS Standards Advisory Number 3. http://www.its.dot.gov/DSRC/.

[12] Lee E., Lim A. (2013). An Empirical Study on *Ad Hoc* Performance of DSRC and Wi-Fi Vehicular Communications. Int. J. Distrib. Sens. Netw..

[13] Armstrong L., Fisher W. IEEE 802.11P Wireless Access for Vehicular Environment, Drafts Standard. http://grouper.ieee.org/groups/802/11/.

[14] Campolo C., Cozzetti H.A., Molinaro A., Scopigno R.M. Overhauling ns-2 PHY/MAC simulations for IEEE 802.11p/WAVE vehicular networks.

[15] Soares V.N.G.J., Farahmand F., Rodrigues J.J.P.C. A layered architecture for Vehicular Delay-Tolerant Networks.

[16] Pereira P.R., Casaca A., Rodrigues J.J.P.C., Soares V.N.G.J., Triay J., Cristina C. (2012). From Delay-Tolerant Networks to Vehicular Delay-Tolerant Networks. Commun. Surv. Tutor..

[17] Wischhof L., Ebner A., Rohling H. (2005). Information Dissemination in Self-Organizing Inter Vehicle Networks. IEEE Trans. Intell. Transp. Syst..

[18] Fogue M., Garrido P., Martinez F.J., Cano J.C., Calafate C.T., Manzoni P. (2013). Identifying the Key Factors Affecting Warning Message Dissemination in VANET Real Urban Scenarios. Sensors.

[19] Li C., Zhao C., Zhu L., Lin H., Li J. (2013). Geographic routing protocol for vehicular ad hoc networks in city scenarios: A proposal and analysis. Int. J. Commun. Syst..

[20] Kaiwartya O., Kumar S. Enhanced Caching for Geocast Routing in Vehicular *Ad-Hoc* Networks (ECGR).

[21] Kaiwartya O., Kumar S., Kasana K. Traffic light based time stable geocast (T-TSG) routing for urban VANETs.

[22] Kaiwartya O., Kumar S. (2014). Cache Agent based Geocasting (CAG) in VANETs. Int. J. Inf. Commun. Technol..

[23] Rao R.S., Soni S.K., Singh N., Kaiwartya O. (2014). A Probabilistic Analysis of Path Duration Using Routing Protocol in VANETs. Int. J. Veh. Technol..

[24] Kaiwartya O., Kumar S. Geocasting in vehicular *adhoc* networks using particle swarm optimization.

[25] UUCP Mapping Project http://tools.ietf.org/pdf/draft-barber-uucp-project-conclusion-05.pdf.

[26] Network Working Group DNS Encoding of Geographical Location. https://www.ietf.org/rfc/rfc1712.txt.

[27] Network Working Group IMAP URL Scheme. http://www.isi.edu/in-notes/rfc2192.txt.

[28] Finn G.G. (1987). Routing and Addressing Problems in Large Metropolitan Scale Internetworks, ISI Research Report.

[29] Kaiwartya O., Kumar S. Geocast Routing: Recent Advances and Future Challenges in Vehicular *Adhoc* Networks.

[30] Maihofer C. (2004). A survey of geocast routing protocols. Commun. Surv. Tutor..

[31] Stojmenovic I., Ruhil A.P., Lobiyal D.K. (2006). Voronoi diagram and convex hull based geocasting and routing in wireless networks. Wirel. Commun. Mob. Comput..

[32] Tsiachris S, Koltsidas G., Pavlidou F. (2013). Junction-Based Geographic Routing Algorithm for Vehicular *Ad hoc* Networks. Wirel. Pers. Commun..

[33] Raw R.S., Das S. (2013). Performance analysis of P-GEDIR protocol for vehicular *adhoc* network in urban traffic environments. Wirel. Pers. Commun..

[34] Fonseca A., Vazao T. (2012). Applicability of position-based routing for VANET in highways and urban environment. J. Netw. Comput. Appl..

[35] Bilal S.M., Bernardos C.J., Guerrero C. (2013). Position-based routing in vehicular networks: A survey. J. Netw. Comput. Appl..

[36] Tripp-Barba C., Urquiza-Aguiar L., Igartua M.A., Rebollo-Monedero D., de la Cruz Llopis L.J., Mezher A.M., Aguilar-Calderón J.A. (2014). A Multi-metric Map-Aware Routing Protocol for VANETs in Urban Areas. Sensors.

[37] Yang Q., Lim A., Li S., Fang J., Agrawal P. (2010). ACAR: Adaptive connectivity aware routing for vehicular *adhoc* networks in city scenarios. Mob. Netw. Appl..

[38] Galaviz-Mosqueda G.A., Aquino-Santos R., Villarreal-Reyes S., Rivera-Rodríguez R., Villaseñor-González L., Edwards A. (2012). Reliable Freestanding Position-Based Routing in Highway Scenarios. Sensors.

[39] Cha S., Lee K., Cho H. (2012). Grid-Based Predictive Geographical Routing for Inter-Vehicle Communication in Urban Areas. Int. J. Distrib. Sens. Netw..

[40] Liu C., Chigan C. (2012). RPB-MD: Providing robust message dissemination for vehicular *adhoc* networks. Ad Hoc Netw..

[41] Shivshankar S., Jamalipour A. (2014). Spatio-temporal multicast grouping for content-based routing in vehicular networks: a distributed approach. J. Netw. Comput. Appl..

[42] Soares V.N.G.J.S., Rodrigues J.J.P.C, Farid F. (2014). GeoSpray: A geographic routing protocol for vehicular delay tolerant networks. Inf. Fusion.

[43] Chen Y.S., Lin Y.W. (2012). A mobicast routing protocol with carry-and-forward in vehicular *adhoc* networks. Int. J. Commun. Syst..

[44] Cheng P.C., Lee K.C., Gerla M., Harri J. (2010). GeoDTN + Nav: Geographic DTN routing with navigator prediction for urban vehicular environments. Mob. Netw. Appl..

[45] Vandenberghe W., Velde E.V., Blondia C., Moerman I., Demeester P. (2012). Vehicular *ad hoc* networking based on the incorporation of geographical information in the IPv6 header. EURASIP J. Wirel. Commun. Netw..

[46] Li F., Zhao L., Fan X., Wang Y. (2012). Hybrid Position Based and DTN Forwarding for Vehicular Sensor Networks. Int. J. Distrib. Sens. Netw..

[47] Felice M.D., Bedogni L., Bononi L. (2013). Group communication on highways: An evaluation study of geocast protocols and applications. Ad Hoc Netw..

[48] Chan W. (2010). Evaluation of Routing Protocols in VANETS: Concepts, Evaluation Methods, Performance Analysis.

[49] Yang J., Fei Z. (2013). Broadcasting with Prediction and Selective Forwarding in Vehicular Networks. Int. J. Distrib. Sens. Netw..

[50] Artimy M.M., Robertson W., Phillips W.J. Assignment of dynamic transmission range based on estimation of vehicle density.

[51] Artimy M. (2007). Local Density Estimation and Dynamic Transmission-Range Assignment in Vehicular *Ad Hoc* Networks. IEEE Trans. Intell. Transp. Syst..

[52] Goldsmith A. (2005). Wireless Communications.

[53] Erceg V., Greenstein L.J., Tjandra S.Y., Parkoff S.R., Gupta A., Kulic B., Julius A.A., Bianchi R. (2006). An empirically based path loss model for wireless channels in suburban environments. IEEE J. Sel. Areas Commun..

[54] Rappaport T.S. (2002). Wireless Communications: Principles and Practice.

